# Identification of early stage and metastatic prostate cancer using electrochemical detection of beta-2-microglobulin in urine samples from patients

**DOI:** 10.1038/s41598-023-37886-4

**Published:** 2023-06-30

**Authors:** Hashmatullah Nasimi, Jonna Skov Madsen, Ahmed H. Zedan, Anders Malmendal, Palle Jörn Sloth Osther, Fatima AlZahra’a Alatraktchi

**Affiliations:** 1 Department of Science and Environment, Roskilde University, Universitetsvej 1, 4000 Roskilde, Denmark; 2 Department of Biochemistry and Immunology, Lillebaelt Hospital, University Hospital of Southern Denmark, Vejle, Denmark; 3 Department of Oncology, Lillebaelt Hospital, University Hospital of Southern Denmark, Vejle, Denmark; 4 Department of Urology, Lillebaelt Hospital, University Hospital of Southern Denmark, Vejle, Denmark; 5 Department of Regional Health Research, University of Southern Denmark, Odense, Denmark

**Keywords:** Biotechnology, Cancer, Biomarkers, Oncology, Electrochemistry

## Abstract

To improve prostate cancer (PCa) diagnosis, it is imperative to identify novel biomarkers and establish effective screening techniques. Here, we introduce electrochemical biosensing of β-2-Microglobulin (β2M) in urine as a potential diagnostic tool for PCa. The immunosensor is composed of a screen-printed graphene electrode coated with anti β2M antibodies. The sensor is capable of detecting the protein directly in urine without any sample pretreatment within 45 min including sample incubation and a lower limit of detection of 204 µg/L. The sensor demonstrated a significant difference in the β2M-creatinine ratio in urine between control and both local- and metastatic PCa (mPCa) (P = 0.0302 and P = 0.0078 respectively), and between local- and mPCa (P = 0.0302). This first example of electrochemical sensing of β2M for the diagnosis of PCa may set the stage for an affordable, on-site screening technique for PCa.

## Introduction

Prostate cancer (PCa) is the second most common cancer in men^1^. Worldwide, PCa accounts for 7.3% of total cancer cases and 3.8% of cancer-related deaths^1^. The lack of more specific diagnostic and better prognostic biomarkers for significant PCa often leads to over-diagnosis and overtreatment^2^. Thus, there is a critical need to develop better non-invasive diagnostic methods with new biomarkers that are more sensitive and specific for PCa.β-2-Microglobulin (β2M) is a glycoprotein that is synthesized by all nucleated cells. It consists of 99 amino acids and forms a small subunit of major histocompatibility class I (MHC I)^3^. The noncovalent association of β2M with the α-chain of MHC class I molecules and no direct attachment to the cell membrane gives rise to free β2M in the body fluids due to intercellular release^4^. Free and soluble β2M in the blood is mainly linked to kidney diseases, although it is suspected to be linked to other diseases. The β2M in body fluids such as blood and cerebrospinal fluids have been hypothesized to potentially play a role in the detection of various diseases, such as diabetes, heart disease, and myeloma, and more generally immunodeficiency in the innate and adaptive immune system^3,5^. Furthermore, recent studies demonstrate that β2M is associated with the regulation of survival, proliferation, and apoptosis in cancer cells, and therefore targeting β2M signaling pathways provides a new strategy for cancer therapeutics^4^. Heretofore, methods used to detect β2M include radioimmunoassay^6^, western blotting^7^, enzyme-linked immunosorbent assay (ELISA), and matrix-assisted laser desorption/ionization/ time-of-flight (MALDI-TOF)^3,5,8^. However, most of the methods available in centralized laboratories, are time-consuming, costly, and non-portable^3,5^. Thus, there is a need for a cheap, fast, and simple detection technique that may be used for fast bedside screening. Electrochemical biosensors hold great promise in the biomedical area due to the direct conversion of the physical interaction with the sensor to electronic signal^9–11^. The enhanced specificity, sensitivity, and cost-effectiveness make them ideal for rapid point-of-care detection of diseases at the bedside^10^. Earlier studies have demonstrated that detection of β2M with biosensors is possible^3,5,12–14^ (Table 2). Rizwan et al.^3^ fabricated an electrochemiluminescence immunosensor based on gold nanoparticles-doped/carbon nano-onions chitosan nanocomposite modified cadmium selenide quantum (QDs-SPE/AuNPs/CNOs-CS) for detection of β2M. This sensor demonstrated the potential to detect β2M in serum and urine samples based on recovery studies^3^. Maity et al. demonstrated the quantification of β2M in human tears. The sensor consists of aqueous gold nanoparticles coated with anti-β-2-microglobulin antibodies using a linker. The suspension of AuNPs shows a specific coloration when the interaction with light occurs^5^. Liu et al.^12^ developed an electrical sandwich immunoassay based on different inorganic nanocrystal tracers.

**Figure 1:**
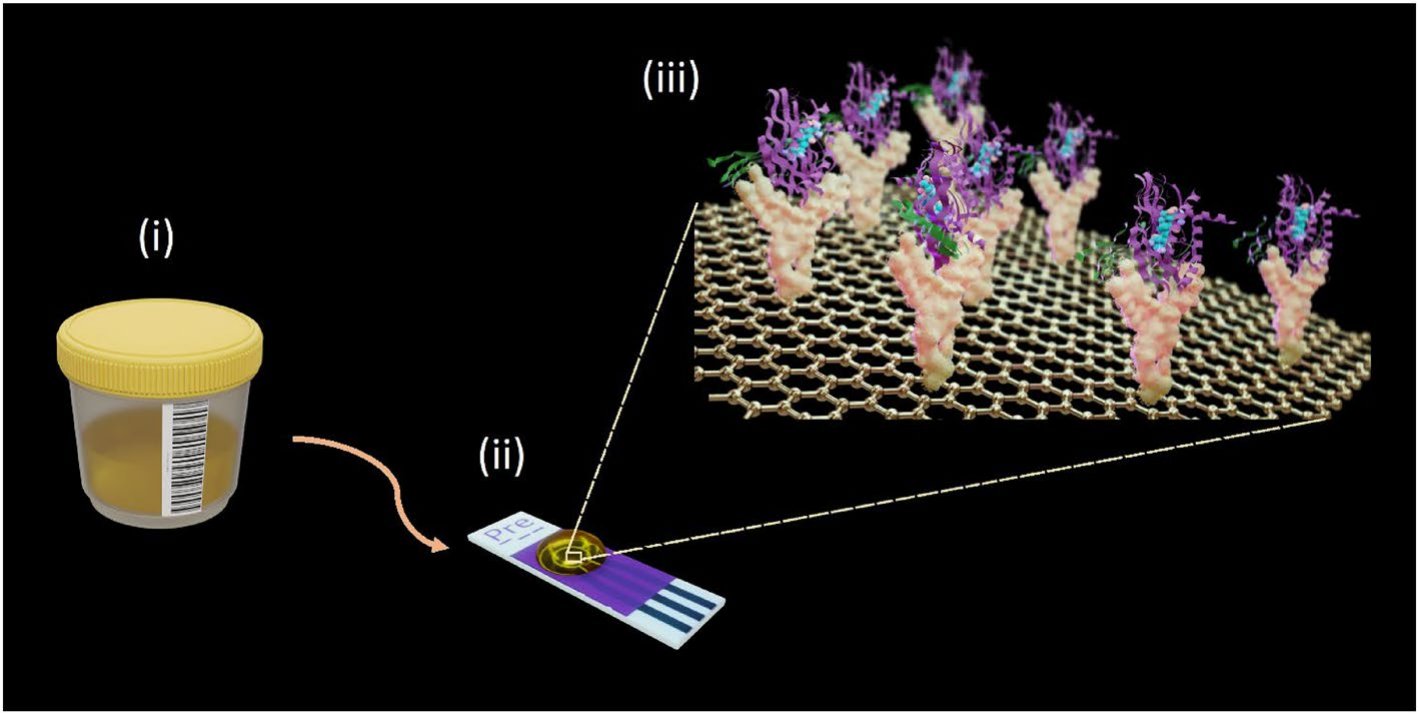
Concept of urinary β-2-microglobulin detection using an electrochemical immunosensor composed of graphene working electrode with anti-β-2-microglobulin functionalization. (i) A urine sample is applied on an (ii) electrochemical scree-printed sensor. (iii) The working electrode of the sensor is functionalized with β-2-microglobulin-specific antibodies.

Here we present an immunosensor composed of screen-printed graphene electrodes coated with anti-β-2-microglobulin (anti-β2M) antibodies using a linker, 1-pyrene butyric acid N-hydroxysuccinimide ester (PBASE), a concept shown in Fig. 1. The introduction of PBASE, antibodies, and bovine serum albumin (BSA) onto the sensor surface result in a reduction in current due to the obstruction of electron transport towards the electrode surface. As a result, when β2M protein binds to the electrode, the expected signal output is diminished in comparison to a sample without protein binding. Our measurements show a significant difference in the levels of β2M between 5 patients referred with a suspicion of PCa, but prostate biopsies showed no evidence for cancer (controls) and 10 PCa patients (5 with local PCa and 5 with metastatic PCa). To the best of our knowledge there have been no reports on electrochemical immunosensors for use in point-of-care detection of β2M in PCa patients and our method may be a breakthrough in the field of diagnostic tools for PCa.

## Material and methods

### Study population and sample collection

The research project received approval from the Southern Denmark Regional Committee on Health Research Ethics (Reference No.: S-20210085) and utilized urine samples from the PerPros Biobank (Reference No.: 18/11174), Department of Urology at Vejle Hospital. All methods were carried out in accordance with relevant guidelines and regulations. Informed consent was obtained from all subjects prior to sample collection. A total number of 15 male individuals were included, 5 patients referred with a suspicion of PCa, whose biopsies were without any evidence for PCa (controls), and 10 PCa patients (five diagnosed with local PCa and five have de novo metastatic PCa). Urine samples from all PCa patients were collected before initiation of androgen deprivation therapy (ADT) and were obtained at any time during the day with no manipulation. The urine samples were collected from patients with normal renal function. All samples were stored at − 80 °C. Table 1 gives baseline clinical information’s about the patients included in this study.

**Table 1:** Demographic characteristics of the patients based on age, PSA, earlier cancer, (Gleason score (GS), and T-stage.

Patient	Age (Years)	Baseline PSA	Earlier cancer	Gleason score	T-stage	Control	Local PCa	mPCa
1	67	6.3	No			x		
2	76	3.9	No			x		
3	70	3.9	No			x		
4	63	3.5	No			x		
5	70	4.8	No			x		
6	64	3.7	No	6 (3 + 3)	N/A		x	
7	68	4.3	No	6 (3 + 3)	N/A		x	
8	67	8.3	No	6 (3 + 3)	T1		x	
9	78	8.8	No	6 (3 + 3)	T1		x	
10	56	3.6	No	6 (3 + 3)	N/A		x	
11	91	45	No	9 (4 + 5)	T2			x
12	79	–	No	9 (4 + 5)	T3			x
13	70	–	No	7 (4 + 3)	T3			x
14	91	135	No	9 (4 + 5)	T3			x
15	66	38	No	7 (4 + 3)	T2			x

### Electrochemical detection of β2M

β2M was quantified using screen-printed graphene sensors from PreDiagnose (Karlslunde, Denmark) featuring a three-electrode design, which comprised a graphene working electrode (WE), a counter electrode (CE), and a graphene reference electrode (RE). The data acquisition was performed using PS-trace 5.8 software (PalmSens BV, Houten, The Netherlands) and the potentiostat Palmsense 4 device (PalmSens BV, Houten, The Netherlands).

### Functionalization of graphene sensors

To enable binding between the anti-β2M antibody and the graphene-based sensor, a surface modification step is employed, which allows for the attachment of the anti-β2M antibody to the graphene surface via a specific linker. Graphene screen-printed sensors were modified according to the protocol developed by Mojsoska et al.^15^. Initially, the Pyrenebutyric acid N-hydroxysuccinimide ester (PBASE, #63520) from Lumiprobe (Hannover, Germany) was dissolved in a 1:5 volume/volume ratio of dimethylformamide (68-12-2, Sigma-Aldrich) and methanol (67-56-1, Sigma-Aldrich). The PBASE solution was then diluted with pure methanol to attain a concentration of 2.5 mM.

The working electrode was coated with a 50 µL layer of the 2.5 mM PBASE solution and incubated for 2 h at room temperature (RT) within a sealed petri dish to prevent evaporation. Subsequently, the sensors underwent washing with pure methanol followed by deionized water and drying with nitrogen. The linker-modified graphene sensors were incubated overnight at RT with 10 µL of anti-β2M antibody (#GTX42680, GeneTex) diluted 80× in Phosphate-buffer solution (PBS). Following the washing and drying phase, the sensors underwent incubation with a 50 µL PBST solution (comprised of PBS and 0.05% Tween-20) containing 1% BSA at room temperature, to hinder non-specific binding. The sensors underwent washing with deionized water and rinsing in PBST (at 37 °C) on a magnetic stirrer at 500 rpm, followed by drying with a gentle flow of nitrogen. Subsequently, the electrochemical signal of the functionalized graphene sensor was measured.

### Characterization of carbon electrochemical sensors

Characterization of the graphene electrode at each stage of functionalization was accomplished by cyclic voltammetry (CV). All electrochemical measurements were carried out using a 2.5 mM ferriferrocyanide solution in PBS and measured against a graphene reference electrode. The CVs were performed from − 1.1 to 1.0 V with scan rates between 0.01 and 0.20 V/s.

### Detection of β2M

β2M in the samples was measured after incubating the functionalized sensors with 10 µL of the sample for 45 min at 37 °C. The sensors underwent washing with deionized water and rinsing in PBST (at 37 °C) on a magnetic stirrer at 500 rpm, followed by drying with nitrogen. The electrochemical measurement of β2M was performed using Square Wave Voltammetry (SWV). SWV was conducted with the following settings: amplitude: 0.25 V; frequency 15.0 Hz and the current ranges from 1 nA to 1 mA, E_begin_ − 0.75 V, and E_end_ 0.8 V. A new sensor was used for each measurement.

### Stability of β2M

To examine the β2M degradation pattern, a stability examination was carried out. Urine samples containing 300 µmol/L of β2M were stored at two temperatures, − 80 °C and 4 °C. On day 0 and day 10, triplicate electrochemical measurements were performed. The signals obtained from the stability experiment were then normalized based on the measurement taken on day 0.

### Recovery test

The sensor was used for the determination of β2M in urine samples to evaluate the efficiency of the proposed method. The urine samples from three different individuals were measured in triplicates before and after spiking with a known quantity of 300 µg/L β2M. The obtained peak heights were converted to concentrations using the standard curve in Fig. 4b.

### Data analysis

The signal induced by β2M binding to the sensor surface was obtained by subtracting the background signal from the analyte signal in PS. Trace 5.8. After subtraction of the background, the peak height was found. The average peak height for each patient was presented. The limit of detection (LOD) was calculated as 3 times the standard deviation of the lowest concentration signal divided by the slope of the calibration curve. Unpaired T-tests with Welch’s test were performed using Graphpad.

## Results

### Characterization of functionalization steps

**Figure 2:**
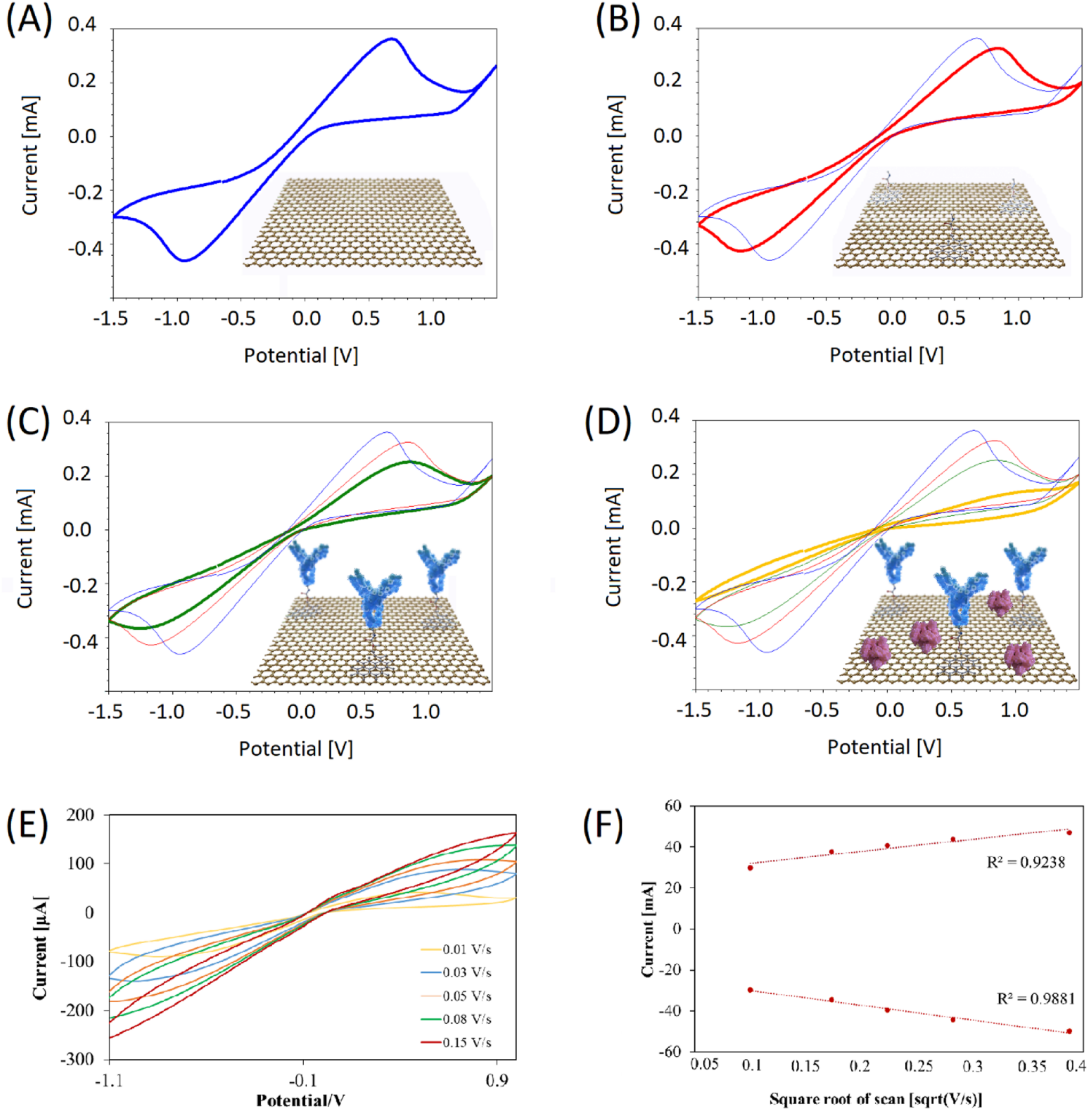
Characterization of functionalization steps and sensor reliability. (**A**) Cyclic voltammograms of (**A**) a bare electrode with no sensor modification, (**B**) an electrode with immobilized linker molecules, (**C**) an electrode with immobilized antibodies, (**D**) an electrode with bovine serum albumin (for blocking non-specific binding) shows the decrease in the current due to the blocking of electron transport towards the electrode surface. (**E**) Cyclic voltammograms at increasing scan rates using the fully functionalized sensor. (**F**) The Randle–Sevcik plot of the peak currents extracted from the cyclic voltammograms versus the square root of the scan rate, deeming the sensor compliant with analytical detection of concentration perturbations. All measurements are conducted in ferriferrocyanide.

To assess each functionalization step, CV measurements of 2.5 mM ferriferrocyanide buffer were performed as seen in Fig. 2A–D. The introduction of PBASE, antibodies, and BSA on the surface leads to a decrease in the current due to the blocking of electron transport towards the electrode surface. The performance and reversibility of the graphene sensor after completed functionalization were evaluated by CVs of 2.5 mM ferriferrocyanide at different scan rates (Fig. 2E). The results showed symmetrical voltammograms and the half-wave potential for a reversible system (each voltammogram) was calculated according to the standard equation (*E*_0_ = (*E*_pa_ + *E*_pc_)/2), yielding a value of − 0.15 ± 0.0245 V vs. the reference electrode. The Randle–Sevcik plot indicated that the sensor is suitable for analytical detection, as the peak current of anodic and cathodic signals are proportional to the square root of the scan rates (Fig. 2F).

### Matrix effect on electrochemical signal

**Figure 3:**
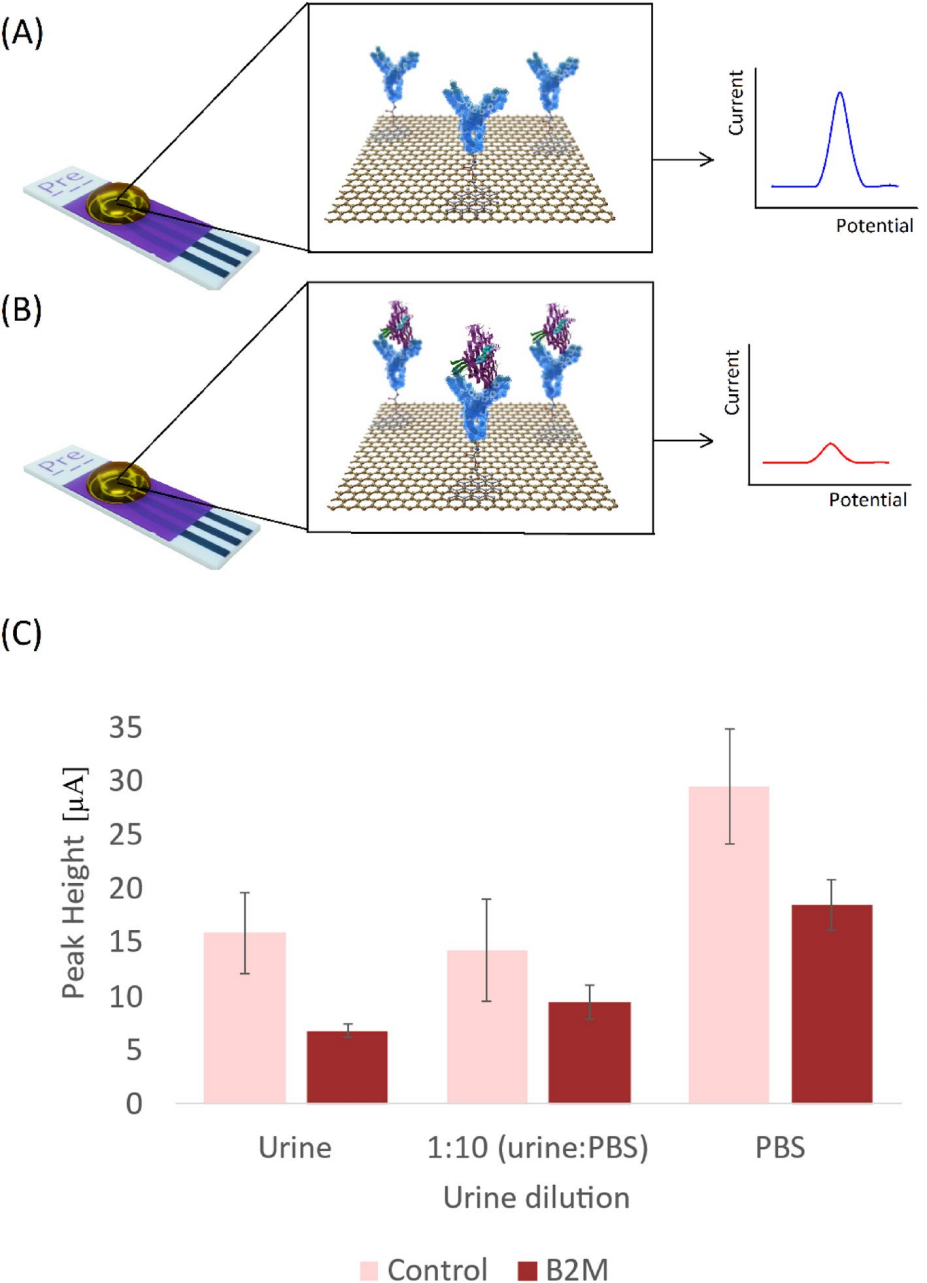
Matrix effect on an electrochemical signal using functionalized electrodes. (**A**) The absence of β2M in the sample will result in a high peak height, (**B**) binding of β2M to the electrode will result in an insulating layer which results in a lower peak height. (**C**) Peak heights of urine, 1:10 (urine: PBS), and pure PBS with and without 300 µg/L β2M.

If the β2M protein is bound to the electrode, the anticipated signal output is suppressed compared to a sample with no protein binding (Fig. 3A,B). The effect of the urine matrix on the signal was characterized by measuring the signal with and without 300 µg/L β2M spiking in unmodified urine, in 1:10 (urine: PBS), and in pure PBS (Fig. 3C). We found that the difference in electrochemical signal between urine with and without 300 µg/L β2M was approximately 57% while the difference in signal was 34% and 37% for 1:10 (urine dilution) and pure PBS, respectively. This suggested that no dilution of urine samples was needed.

### Direct quantification of β2M in urine

**Figure 4:**
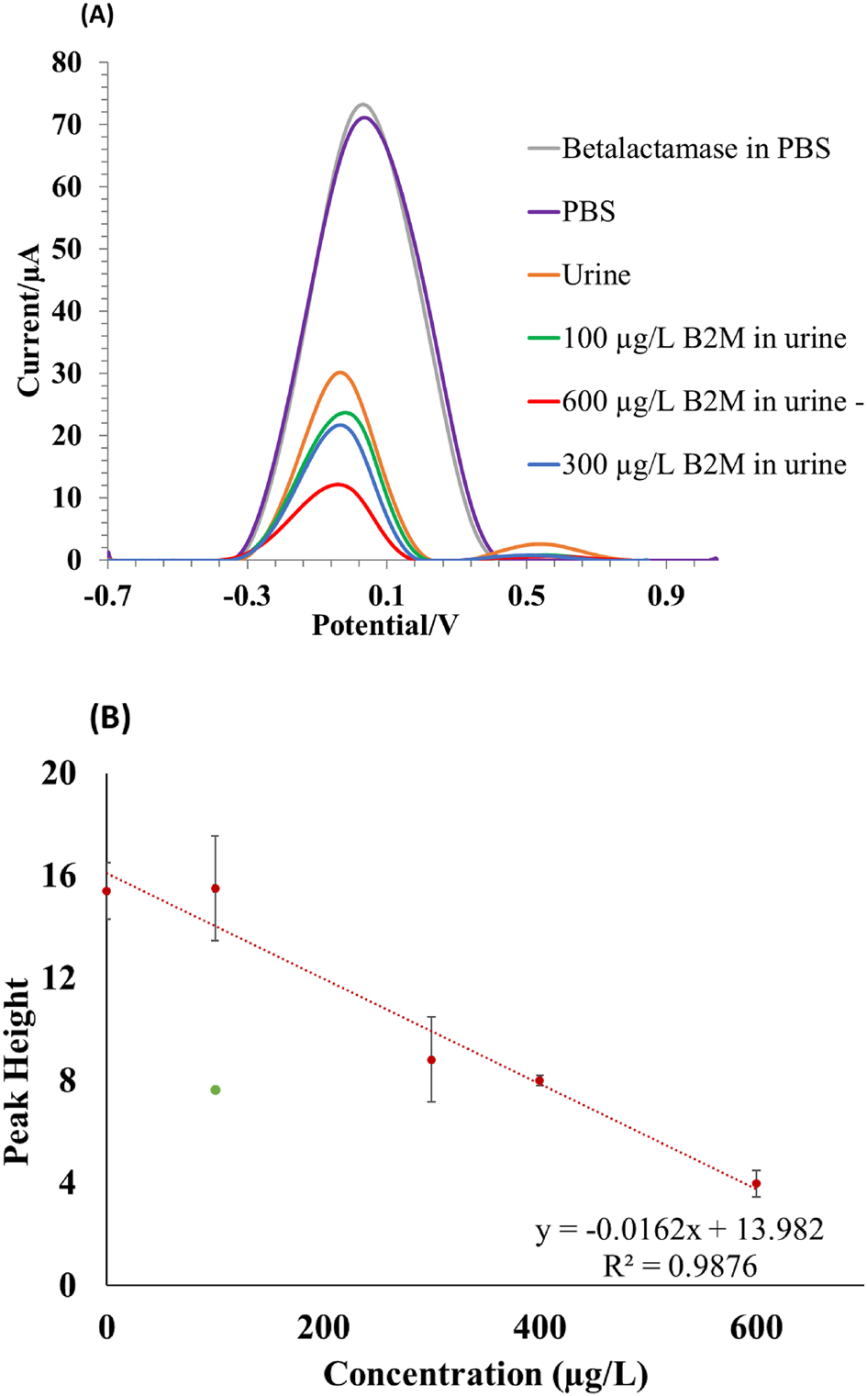
Standard curve for β2M measurements. (**A**) Square wave voltammograms of increasing β2M concentration in urine using modified graphene sensors. (**B**) A standard curve with an inverse correlation between added β2M concentration and peak height measured in urine. The green dot in the graph is the outlier.

The modified graphene electrodes were used to detect β2M in urine at SWV scans from − 0.75 to 1.0 V and the β2M peak appeared at 0.1 V (Fig. 4a). Square wave voltammograms of spiked urine samples show decreasing peak heights with increasing β2M concentration, which could indicate a saturation of the available binding sites. Another possible explanation could be that the effectiveness of antibodies to form immune complexes is impaired due to high levels of either antibody or antigen. Figure 4A shows that pure PBS and Betalactamase (control-protein) in PBS resulted in the highest peak heights due to no β2M present in the samples. The peak width of a peak in a voltammogram usually corresponds to the kinetics of the redox process. Changes in the peak width may happen when the surface area changes due to e.g., incubation with analytes of different matrices. Our data consistently show that the peak width differs depending on the matrix of the analyte, thus measurements conducted in urine have a narrower peak width than the measurements in PBS. The difference in signal between blank urine and pure PBS could be due to that urine naturally contains around 300 µg/L β2M. Despite various urine controls from different healthy men, the observed signal levels were consistent and as expected. Therefore, the data suggest that no interference is observable. The increasing concentrations of added β2M (0–600 µg/L) were measured in otherwise identical urine samples and followed a linear correlation with a slope of − 0.0162 µA/µg/L and a regression coefficient of 0.9876 (Fig. 4b). An outlier, represented by a green dot in the graph, was identified and could be attributed to a scratched sensor. It was subsequently removed and treated as a separate measurement. The LOD was calculated to 204 µg/L in urine as 3 times the standard deviation of the lowest concentration signal divided by the slope of the calibration curve. The graphene-modified electrodes can detect β2M below the normal concentration range in urine which is reported to be 230–300 µg/L^16^.

### Stability of β2M detection

**Figure 5:**
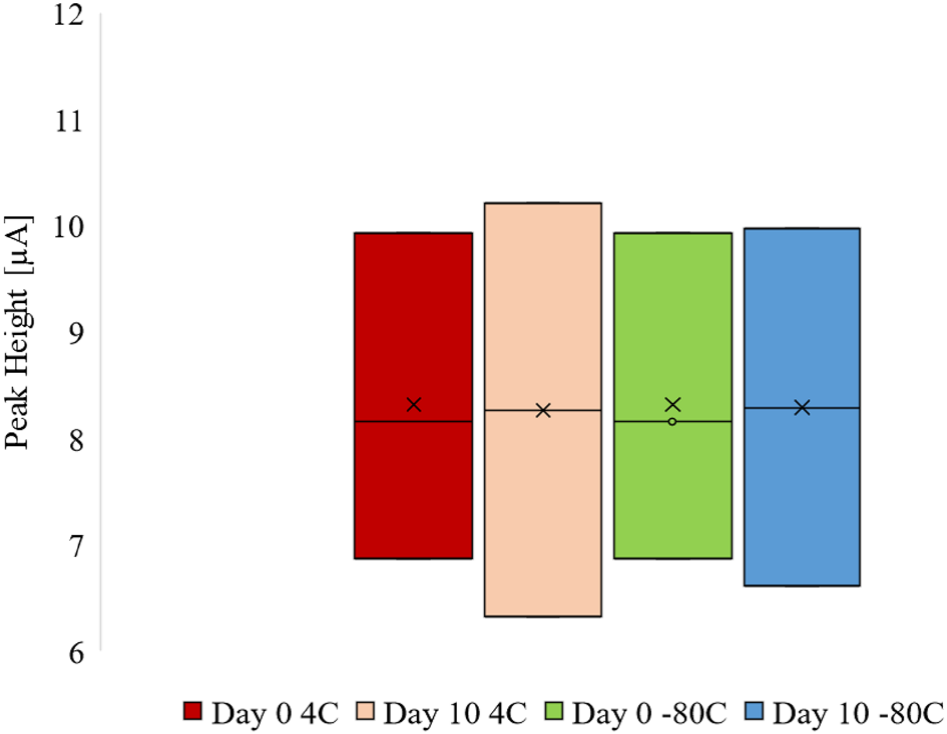
β2M stability test: β2M in urine was stored under different temperature conditions for a period of 10 days. The boxes represent the initial concentration of β2M, which was 300 µmol/L, at Day 0, as well as the recovered concentration at Day 10 for storage conditions at 4 °C and − 80 °C. The test demonstrated that β2M was stable under all conditions during the test period.

To ensure that β2M is stable despite the storage of samples under different temperatures, a stability test was performed in urine from one control patient in triplicates, by measuring the signal of a constant concentration at fixed temperatures for a period of 10 days. As patients’ urine samples are regularly stored at 4 °C and − 80 °C, the stability of the electrochemical signal of β2M detection was investigated after storage at these temperatures. Figure 5 shows the recovered concentration as a function of time for samples with 300 µmol/L of β2M. After 10 days no significant degradation was observed.

### Recovery of β2M in spiked urine samples

The functionalized graphene sensor was successfully applied for the detection of β2M levels in human urine samples in recovery investigations (Table 2). The urine samples from three different individuals were measured before and after spiking with a known quantity of 300 µg/L. The obtained peak heights were converted to concentrations using the standard curve in Fig. 4b. All samples contained between 239 and 275 µg/L β2M which is within the naturally occurring levels in healthy individuals (230–300 µg/L). The average recovery rate was 102 ± 7%.

**Table 2:** Recovery of β2M in spiked urine samples. Samples were spiked with 300 µg/L β2M.

Sample	[β2M] before spiking (µg/L)	[β2M] after spiking (µg/L)	Recovery (%)
1	239	542	102
2	264	544	93
3	275	605	110

### Difference in β2M between controls and PCa patients

**Figure 6:**
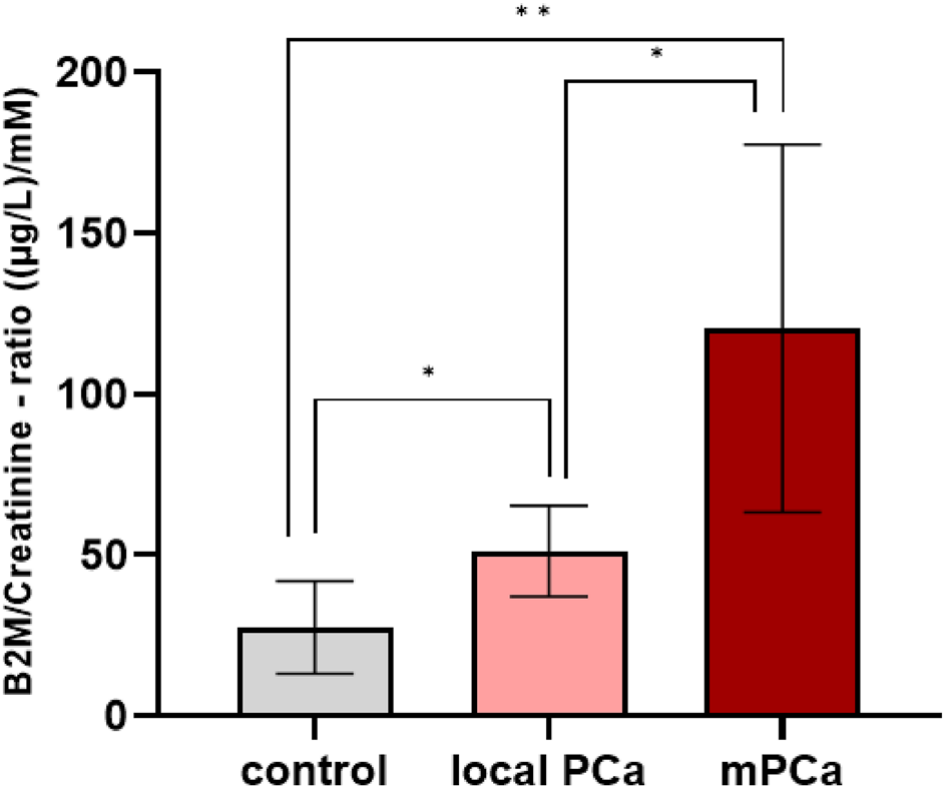
β2M levels in the urine samples for the control group, local PCa, and metastatic PCa. The biosensor demonstrated that there is a significant increase in levels of β2M in urine samples from PCa patients compared to those from the control group, *P ≤ 0.05, **P ≤ 0.01.

The modified graphene electrodes were finally applied to investigate the difference in β2M levels between controls and patients with PCa. The β2M to creatinine ratio was measured for all patients included in the study and compared between patient groups (Fig. 6). The data showed significant differences between the control group and both the local PCa (*P* = 0.03) and the metastatic PCa (*P* = 0.008). Also, a significant difference between local PCa and mPCa was observed (*P* = 0.03). A significant difference for the measured β2M levels (not corrected for creatinine) was observed between the controls and PCa group (*P* = 0.009) (Supplementary Fig. 1). Even when we take into account the 7% uncertainty calculated from the recovery experiment, there is still a statistically significant difference between the control and patient groups. In one of the control samples β2M was measured to be197 µg/L, which is under the LOD.

### Other biosensors for β2M quantification

Table 3 gives an overview of the previously reported biosensors for β2M detection in comparison to this work. The comparison is based on the sensing materials, detection methods, limits of detection, media, and recovery.

**Table 3:** Comparison of performance of this work with previously reported β2M sensors.

Material of sensor	Detection method	LOD in media	Media	Recovery in real samples	References
QDs-SPE/AuNPs@CNOs-CS	ECL	1 fg/mL	Tris-buffer	92–96%	^3^
Antigen–Antibody-DTSP-AuNP	Optical	100 fg/mL	PBS	67–136%	^5^
Colloidal nanocrystal tags	SWV	0.0001 fg/mL	PBS	–	^12^
Series S carboxymethylated	Surface plasmon resonance	1.3 × 10^−5^ fg/mL	500–1000-fold diluted serum in HBS-EP +	–	^17^
Quartz crystals microbalance	Piezoelectric	3 × 10^−6^ fg/mL	PBS	90–110%	^18^
Grating coupler	HeNe-laser	5 × 10^9^ µg/mL	PBS	–	^19^
Antigen–antibody-Gr	SWV PBS	2.4 × 10^8^ fg/mL 5.5 × 10^7^ fg/mL	Urine PBS	93–110%	This work

*ECL* electrochemiluminescence, *SWV* squarewave voltammetry, *LOD* limit of detection, *GCE* glassy carbon electrode, *Gr* graphene, *DTSP* n-Hydroxysuccinimide ester, *HBS-EP* + 10 mM HEPES pH 7.4, 150 mM NaCl, 3 mM EDTA, 0.05% (v/v) P20.

Rizwan et al. fabricated an electrochemiluminescence (ECL) sensor and demonstrated very good sensitivity towards β2M in buffer solution^3^. Maity et al. demonstrated quantification of β2M by an immunosensor, however, the recovery percentages of β2M in urine with this sensor were very varying^5^. The mentioned sensors are the most sensitive towards β2M in buffer solutions, but they are based on plasmon resonance and ECL. Only one of the listed studies in Table 2 has developed an electrochemical immunosensor for the detection of β2M^12^. Liu et al. reported an immunoassay for measurements of multiple proteins based on inorganic nanocrystal tracers^12^. In this immunoassay, the antigens are captured by magnetic beads conjugated with antibodies. The antigens are then detected by reactions with nanocrystal-labeled secondary antibodies and finally dissolution of the nanocrystals occurs, and electrochemical detection is possible^12^. Liu et al.^12^ have not presented measurements in real samples. The current work demonstrated electrochemical measurements of β2M directly in patient urine samples with LOD at the lower end of the physiologically relevant concentration range and without any pretreatment of the patient sample.

## Discussion

Increased levels of β2M have been reported for different cancer types^20–23^. Bataille et al.^23^ showed a significant positive correlation between β2M and myeloma stage. In the PCa population, Zhang et al.^24^ demonstrated that serum β2M was significantly increased in PCa patients compared to benign prostatic hyperplasia (BPH) or normal controls. Furthermore, urinary β2M was reported to be elevated in 74% of advanced PCa patients compared to healthy subjects using radioimmunoassay^20,21^. Additionally, elevated urinary β2M levels in PCa patients with bone metastasis were significantly associated with shorter overall survival^21^. These results suggest a role of β2M in both the development and progression of PCa.

The current diagnostic approaches for PCa have limited potential for distinguishing between indolent and aggressive disease^25^. Due to the risk of overtreatment, more specific diagnostic and prognostic biomarkers are needed^26^. In this respect, β2M may have a potential role as an alternative biomarker for PCa. However, there no currently no low-cost and high sensitivity immunosensor available for use in the point-of-care detection of β2M in urine, so this work can be seen as a potential breakthrough in the field of β2M detection of.

When investigating biomarkers in urine, levels of markers are generally normalized to creatinine measurements^21^. Treatment by ADT results in rapid loss of muscle mass far exceeding that of normal aging, which results in reduced creatinine levels compared with normal subjects^21,27^. Therefore, the normalization of β2M levels with creatinine causes an elevation in the β2M to creatinine ratio in patients that have been treated with hormonal therapy^21,27^. The sampling of the patients included in this study was initiated before treatment by ADT. β2M levels cannot be normalized to urine protein levels because evaluation in urine β2M causes a comparable increase in urine protein levels^21^. In addition, β2M is cleared by the kidneys, and its level also reflects renal function^28^.

For validation of the observations made in this study, we wanted to perform ELISA. However, we were not able to quantify β2M in urine samples with direct or sandwich ELISA. Some of the common shortages of conventional ELISA are limited multiplexing options, the necessity for centralized laboratory equipment, and the relatively high sample volumes required^29^. Also, consistency in handling ELISA comes with practice. Another limitation of using ELISA for the detection of β2M is the concentration range of the commercially available ELISA kits. The normal urinary levels of β2M are reported to be 230–300 µg/L^16^. No ELISA kit was found that could detect β2M in this concentration range. In our study, we chose not to dilute the samples due to the challenges associated with achieving an excessively high dilution factor. Instead, we relied on internal electrode performance assessment to ensure the accuracy and reliability of our results. This is a proof-of-concept study, it is important to stress that further experiments are necessary to refine the proposed immunosensor in terms of practical and clinical applicability. To comprehensively evaluate the sensor performance, a larger validation study is required in the future as well as an investigation of the long-term stability, storage conditions, and shelf life of the sensors.

With the new electrochemical method, we have investigated the difference in urinary β2M levels between controls and patients diagnosed with PCa. Several studies have demonstrated that electrochemical detection of β2M is possible, but to the best of our knowledge, this study is the first to present electrochemical measurements of β2M in urine samples from PCa patients.

Considering the effectiveness and low cost of the electrochemical biosensor combined with the obtained data, usage of the biosensor and β2M as supplementary diagnostic tools potentially may improve the diagnostic accuracy in PCa. However, the observed significant differences between the control group and PCa patients need further validation in larger cohort studies.

## Conclusion

This work established proof-of-concept for an electrochemical biosensor for fast and direct detection of β2M in urine. Cyclic voltammetry was used to characterize the electrode functionalization. Using square wave voltammetry, it was possible to detect β2M in the concentration range 204–600 µg/L, covering the physiologically relevant concentrations, in about 45 min. Using this device, we demonstrated a significant difference in urinary β2M levels between controls and PCa patients. A larger sample size is required for the validation of such observation.

## Supplementary Information

Supplementary Information.
