# Supplementary material for: Identification of early stage and metastatic prostate cancer using electrochemical detection of beta-2-microglobulin in urine samples from patients
Source: Sci Rep. 2023 Jun 30;13:10658. doi: 10.1038/s41598-023-37886-4 (PMC10313715; doi:10.1038/s41598-023-37886-4)
Supplement: Supplementary file 1 — Supplementary Information. [file 41598_2023_37886_MOESM1_ESM.docx]

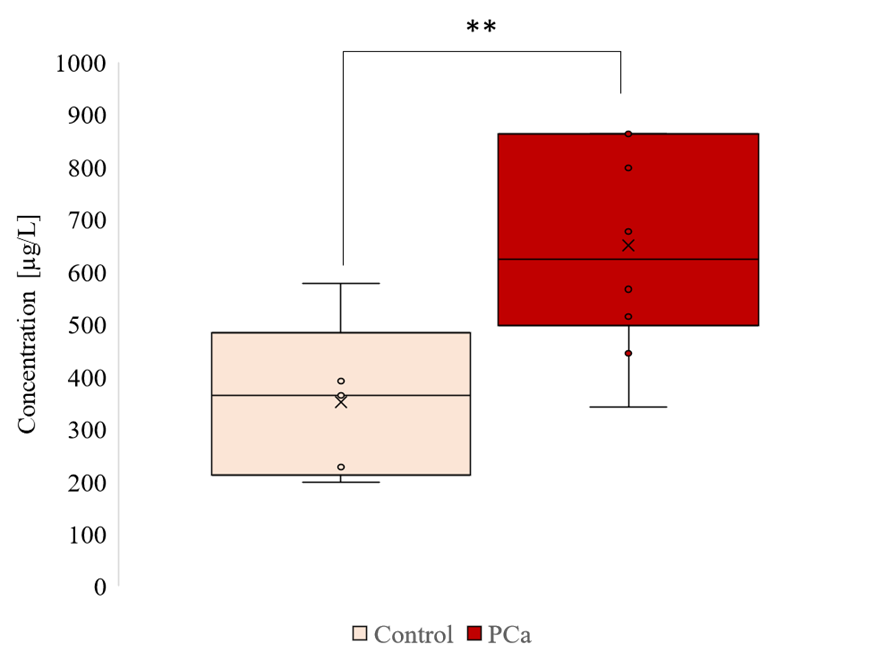


**Supplementary figure 1: β2M levels for control group and PCa patients**. The biosensor demonstrated that there is a significant increase in levels of β2M between the control group and PCa patients, ** P≤0.01.
